# Emergence of Colistin Resistance in Multidrug-Resistant Escherichia coli and Klebsiella Species From Central India: A Sentinel Study With the Detection of mcr-1 From Blood Isolates

**DOI:** 10.7759/cureus.111794

**Published:** 2026-06-30

**Authors:** Pallavi Kumari, Riti Seth, Shubham Nema

**Affiliations:** 1 Microbiology, Netaji Subhash Chandra Bose Medical College, Jabalpur, IND; 2 Research, Netaji Subhash Chandra Bose Medical College, Jabalpur, IND

**Keywords:** central india, colistin resistance, mcr-1, multidrug resistance, surveillance

## Abstract

Background and objectives

Colistin is increasingly reserved for the treatment of severe infections caused by multidrug-resistant (MDR) Gram-negative bacteria; however, emerging resistance is progressively limiting its clinical effectiveness. Although antimicrobial resistance surveillance has expanded in several regions of India, clinical data from Central India remain limited. This study aimed to estimate the prevalence of colistin resistance among MDR *Escherichia coli and Klebsiella *spp. inbloodstream isolates from Central India and to determine the presence of the plasmid-mediated *mcr-1* gene in colistin-resistant isolates.

Methods

This prospective study was conducted at a tertiary care hospital over two years. MDR *E. coli and Klebsiella *spp. isolated from blood cultures were subjected to colistin susceptibility testing using the agar dilution method. Colistin-resistant isolates were further analyzed for the presence of the plasmid-mediated *mcr-1* gene using polymerase chain reaction.

Results

Colistin resistance was seen in 9.67% of *E. coli* and 0.47% of *Klebsiella *spp. of MDR isolates. A single *Klebsiella pneumoniae* isolate harbored the *mcr-1* gene, representing the first such clinical detection from Central India. Of the four phenotypically colistin-resistant MDR isolates, three (75.0%, all *E. coli*) were negative for the *mcr-1* gene by reverse transcription polymerase chain reaction (RT-PCR). The single *mcr-1*-positive isolate, a *Klebsiella pneumoniae*, was recovered from a surgical intensive care unit patient. No other *mcr* variants were tested.

Interpretation and conclusions

The study demonstrates the early emergence of colistin resistance in Central India, predominantly mediated through chromosomal mechanisms, with limited plasmid involvement. These findings emphasize the need for strengthened phenotypic surveillance and targeted infection control measures, particularly in critical care settings.

## Introduction

Over the decades, the widespread use of antibiotics has transformed infectious disease management, but this progress has been accompanied by a steady rise in bacterial resistance against antibiotics. The limited development of new antimicrobial agents has further intensified this challenge, making resistance an increasingly difficult problem [1,2]. Colistin is an essential treatment option for infections caused by *Enterobacterales* that are resistant to carbapenems.

Through intrinsic or acquired carbapenemase-mediated pathways, Gram-negative organisms such as *Klebsiella pneumoniae*, *Pseudomonas aeruginosa*, and *Acinetobacter baumanii *are mostly affected by carbapenem resistance, which has become a persistent global public health concern [3]. The identification of plasmid-mediated mobilized colistin-resistant genes (*mcr*) in 2015 marked a significant shift in the epidemiology of polymyxin resistance, raising concerns regarding their potential for rapid horizontal dissemination [4]. India bears a substantial burden of antimicrobial resistance, contributing significantly to global morbidity and mortality associated with drug-resistant bacterial infections [5,6].

Globally, bacterial antimicrobial resistance is estimated to be associated with 4.95 million deaths annually, representing one of the most significant public health threats of the twenty-first century [7,8]. India contributes disproportionately to this burden given its population size, high antibiotic consumption, and healthcare infrastructure constraints. While surveillance data on carbapenem resistance and colistin resistance are available from northern and southern parts of the country, Central India, particularly Madhya Pradesh, has remained underrepresented in clinical studies [9]. This gap is notable given the region’s high antibiotic consumption and expanding tertiary care services. Earlier investigations from this region have largely focused on animal or environmental reservoirs of *mcr* genes, with limited emphasis on bloodstream infections in hospitalized patients [10-12]. Given the public health implications of transferable colistin resistance, systematic clinical surveillance is essential. Therefore, the current investigation was conducted to ascertain the frequency of colistin resistance among multidrug-resistant (MDR) *Enterobacterales *that were isolated from blood cultures in a central Indian tertiary care hospital and to evaluate the role of *mcr-1* detection in plasmid-mediated resistance.

## Materials and methods

Study design and setting

This prospective observational study was conducted from February 2023 to March 2025 in the Department of Microbiology, Netaji Subhash Chandra Bose Medical College, Jabalpur, a 1,200-bed tertiary care referral hospital in Central India. This study includes laboratory-based isolates. The unit of analysis was the clinical blood-culture isolate, not the individual patient. Patient-level clinical data, including symptoms, comorbidities, treatment regimens, and outcomes, were not collected. Prevalence estimates therefore reflect the frequency of colistin resistance among culture-positive MDR blood-culture isolates processed at this institution during the study period. They may not directly correspond to the incidence of colistin-resistant bloodstream infection in the underlying patient population. Ethical permission regarding this work was obtained from the Institutional Ethics Committee (IEC/2023/7335-171).

Inclusion and exclusion criteria

All MDR strains of *Escherichia coli *and *Klebsiella *spp. isolated from blood cultures of patients admitted to inpatient wards of this institution during the study period were included. MDR was defined as resistance to at least one antibiotic agent in three or more antimicrobial categories.

Exclusion criteria were as follows: (1) other Gram-negative bacteria (*Pseudomonas* spp., *Acinetobacter* spp., *Salmonella* spp.); (2) all susceptible strains of *E. coli* and *Klebsiella* spp.; (3) isolates from outpatients; (4) other clinical specimens (pus, urine, sputum, ascitic fluid, bronchoalveolar lavage, endotracheal aspirate, pleural fluid); and (5) duplicate isolates, defined as a subsequent MDR isolate of the same species obtained from the same patient during the same admission episode with an identical antibiogram pattern. Only the first isolate per episode per patient was retained. A patient presenting with a second bacteremia episode during a separate hospital admission was treated as a new incident case and re-enrolled.

Sample processing and identification

**Figure 1:**
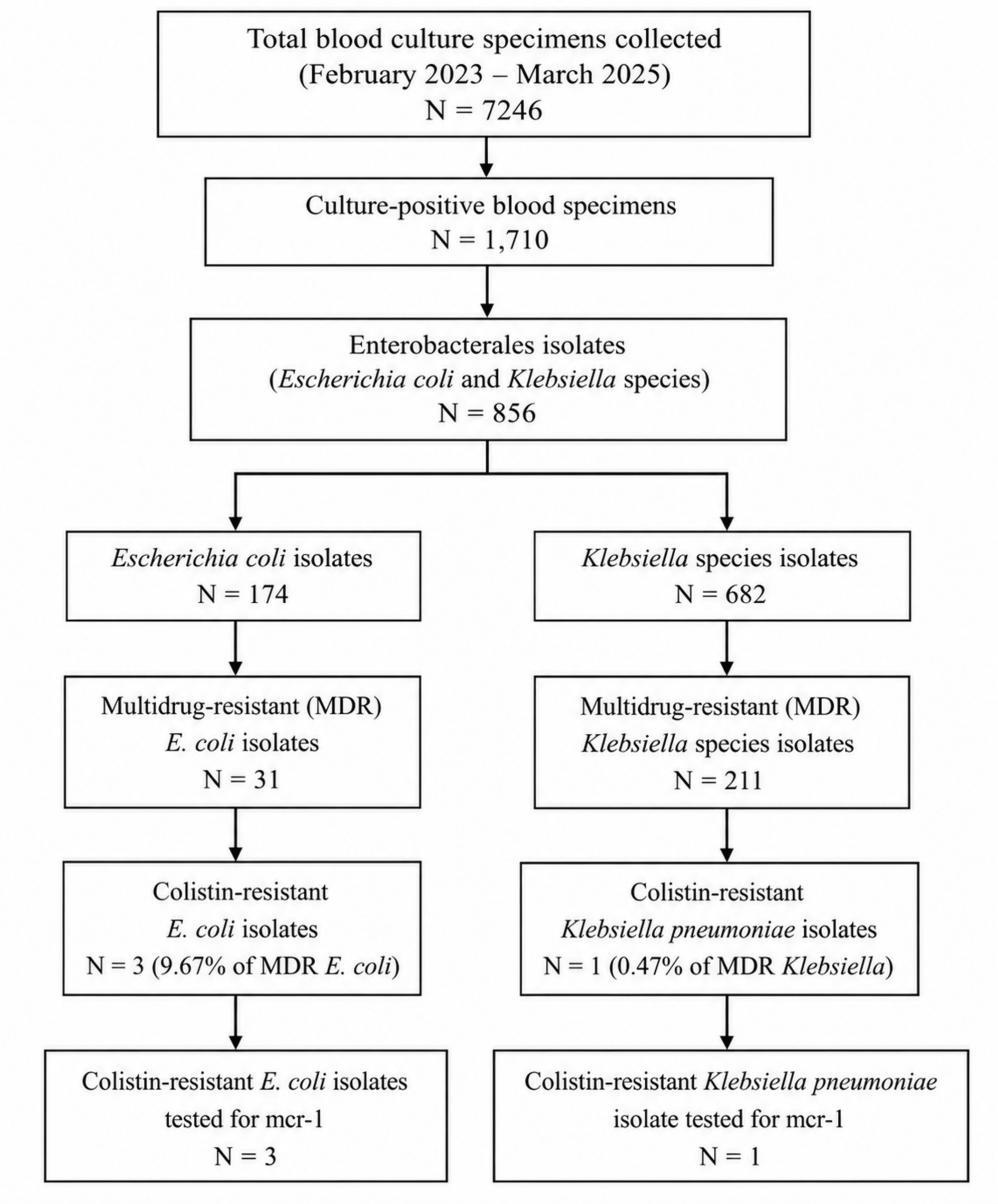
Flow diagram of specimen processing

All blood specimens collected by clinicians were received in the bacteriology section of the Department of Microbiology within 2 hours of collection. All blood specimens were collected from peripheral veins under aseptic conditions and inoculated into two bottles per episode: one for aerobic processing and one for anaerobic processing. The aerobic bottle was used for all subsequent culture steps. Only the first non-duplicate isolate per patient per admission episode was included. Duplicates were identified based on patient identifier, organism species, and antibiogram pattern and were excluded from analysis. Each specimen was inoculated into Brain Heart Infusion (BHI) (HiMedia Laboratories Pvt. Ltd., Mumbai, India) in a ratio of 1:10 (5 mL of blood in 50 mL of BHI broth) using a conventional manual blood culture system and incubated at 37°C for 24 hours. This method was selected because no automated blood culture analyzer was available at this institution during the study period. Following incubation, the specimens were sub-cultured on MacConkey agar and blood agar (both HiMedia Laboratories Pvt. Ltd.), which were incubated overnight at 37°C. After incubation, culture plates were examined for bacterial growth. The isolates obtained were identified based on colony morphology, Gram staining characteristics, and standard biochemical tests such as catalase test, oxidase test, citrate utilization test, urea hydrolysis test, triple sugar iron agar test, indole production test, methyl red test, Voges Proskauer test, and sugar fermentation test (HiMedia Laboratories Pvt. Ltd., Mumbai, India) [13]. Only *E. coli* and *Klebsiella* spp. isolated from blood cultures were included. The flow diagram of specimen processing is shown in Figure 1.

Antimicrobial susceptibility testing

The Kirby-Bauer disk diffusion method was used to test for antimicrobial susceptibility in accordance with the Clinical and Laboratory Standards Institute (CLSI) (M100-S34:2024) [14]. Zone diameter breakpoints of antimicrobials for *E. coli* and *Klebsiella *spp. is given in Table 1. Resistance to at least one agent in three or more classes of antimicrobials is known as multidrug resistance [15]. MDR isolates were tested for colistin susceptibility using the agar dilution method on cation-adjusted Mueller-Hinton agar. Agar dilution was selected as the method for colistin minimum inhibitory concentration (MIC) determination for the following reasons. First, it is endorsed as a reference-tier method by the CLSI (M100-S34:2024). Second, broth microdilution, the formally designated primary reference method, was not available as a validated commercial platform at our institution during the study period. Third, agar dilution is specified in the National Centre for Disease Control (NCDC, India) standard operating procedure for colistin susceptibility testing of Enterobacterales (Version 1, July 2022), which provided the procedural framework for our testing [16]. Colistin (Sigma-Aldrich, St. Louis, MO, USA) was dissolved in distilled water to prepare a stock solution of 1 mg/mL and stored at -20°C until use. Serial dilutions were prepared in cation-adjusted Mueller-Hinton agar (CAMHA; HiMedia Laboratories Pvt. Ltd.) to yield final colistin concentrations ranging from 0.5 to 16 µg/mL. An inoculum equivalent to McFarland 0.5 turbidity standard (~1-2 × 10⁸ CFU/mL) was prepared, further diluted 1:10 in saline, and 1-2 µL was spot-inoculated onto each plate (~5 × 10⁴ CFU/spot) with positive and negative controls. Quality control strains included *E. coli* NCTC 13846 as the positive control and *E. coli* ATCC 25922 as the negative control. Plates were incubated at 37°C for 18-20 hours. MIC was defined as the lowest colistin concentration at which no visible growth was detected. Colistin MIC was determined by the agar dilution method and interpreted using clinical breakpoints (MIC ≥4 µg/mL = resistant; MIC ≤2 µg/mL = intermediate) [16]. Before external validation, internal reading concordance was confirmed for all four resistant isolates. Resistant strains were further validated by the NCDC, New Delhi, by repeated organism identification and MIC testing by the broth microdilution method.

**Table 1:** Zone diameter breakpoints for Escherichia coli and Klebsiella spp.

Antibiotics	Disc potency (µg)	Sensitive	Resistant
Ampicillin (*E. coli *only)	10	≥17	≤13
Amoxicillin-clavulanate	20/10	≥18	≤13
Ceftriaxone	30	≥23	≤19
Ceftazidime	30	≥21	≤17
Tobramycin	10	≥17	≤12
Amikacin	30	≥20	≤16
Tetracycline	30	≥15	≤11
Doxycycline	30	≥14	≤10
Ciprofloxacin	5	≥26	≤21
Levofloxacin	5	≥21	≤16
Imipenem	10	≥23	≤19
Ertapenem	10	≥22	≤18
Meropenem	10	≥23	≤19
Gentamicin	10	≥18	≤14
Co-trimoxazole	1.25/23.75	≥16	≤10

Molecular detection of *mcr-1*

Colistin-resistant isolates were screened exclusively for *mcr-1*, as this is the most globally prevalent plasmid-mediated colistin resistance gene and was the first to be described. Testing for other *mcr* variants (*mcr-2 *through *mcr-10*) was beyond the capacity of the available commercial kit at our institution and is acknowledged as a limitation of this study. Nucleic acid extraction from phenotypically colistin-resistant isolates was done using the Nucleic Acid Extraction Kit v2.0 following the prescribed protocol and thereafter screened for the *mcr-1* gene using real-time polymerase chain reaction using the MCR Detection Kit (Qualitative RT-PCR; Huwel Lifesciences Private Ltd., Hyderabad, India), which contains sequence-specific primers for *mcr-1* [Forward: 5′-CTCGTTGGCTTAGATGACT-3′; Reverse: 5′-AAGTGCGAACATCAGTCC-3′] [17]. Reverse transcription polymerase chain reaction (RT-PCR) cycling conditions were as follows: initial denaturation at 95°C for 15 minutes; 40 cycles of denaturation at 95°C for 10 seconds, and annealing/extension at 60°C for 30 seconds; followed by a melt curve analysis. A cycle threshold (Ct) value of ≤35 was used to define a positive result, as recommended by the kit manufacturer. Each PCR run included a manufacturer-provided positive control and nuclease-free water as a no-template control (NTC) to ensure assay validity and monitor for potential carry-over contamination.

Statistical analysis

Statistical analysis was performed using OriginPro 16 Scientific Data Analysis and Graphing Software (OriginLab Corporation, Northampton, MA, USA). Primarily, two comparisons were planned: (1) MDR prevalence between *Klebsiella* spp. and *E. coli*, tested using Pearson's chi-square with Yates continuity correction; (2) colistin resistance rates between MDR *Klebsiella* spp. and MDR *E. coli*, tested by Fisher's exact test (two-tailed), as all expected cell frequencies were <5. The phenotype-genotype comparison (*mcr-1 *carriage vs colistin resistance status across all MDR isolates) was exploratory. Fisher's exact test was used for this comparison, and the result should be interpreted in the context of the very small event count (n = 4 resistant isolates). All proportions are reported with Wilson score 95% confidence intervals (CIs). A p-value of <0.05 was considered statistically significant.

## Results

Distribution of isolates and MDR prevalence

**Figure 2:**
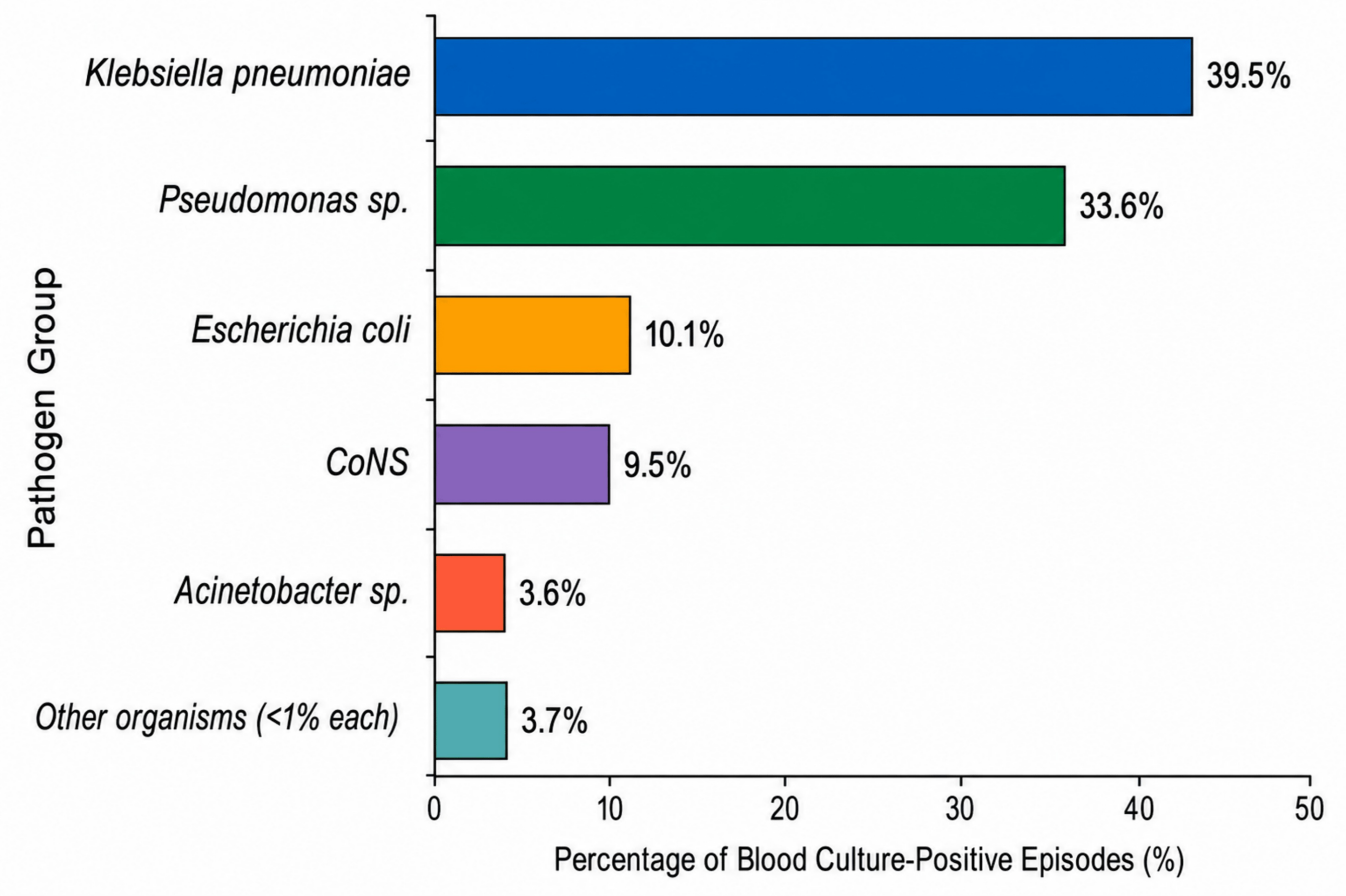
: Distribution of pathogens identified from 1,710 blood culture-positive cases. *Klebsiella pneumonia*e (39.5%), *E. coli* (10.1%), and *Pseudomonas* spp. (33.6%) were the three most frequent isolates. Only *E. coli* and *Klebsiella* spp. were included in the study. CoNS, coagulase-negative staphylococcus

Among 1,710 blood culture-positive specimen, 676/1,710 (39.5%) isolates were *Klebsiella pneumoniae* and 6/1,710 (0.35%) isolates were *Klebsiella oxytoca*, while 174/1,710 (10.1%) isolates were *E. coli*, 576/1710 (33.6%) isolates were *Pseudomonas* spp., 162/1710 (9.4%) isolates were coagulase-negative staphylococcus, 62/1710 (3.6%) isolates were *Acinetobacter* spp., 27/1710 (1.5%) isolates were *Enterococcus faecalis*, 1/1710 (0.05%) isolate were *Enterococcus faecium*, 22/1710 (1.2%) isolate were *Staphylococcus aureus*, and 4/1710 (0.23%) isolate were *Candida* spp. (Figure 2). Only *Klebsiella *spp. (n = 682) and *E. coli* (n = 174; combined n = 856) were included in further analysis. Multidrug resistance was significantly more frequent among *Klebsiella* spp. (211/682, 30.9%) than *E. coli *(31/174, 17.8%) (p <0.001) (Table 2). MDR isolates from positive culture were predominantly recovered from intensive care units (ICUs), specifically the maximum MDR burden found in the gynecology ICU (45/142, 31.7%), followed by medicine ICU (33/152, 21.7%) and surgical ICU (46/294, 16%). Irrespective of individual collection sites, male patients had considerably higher MDR infection rates (59.3%) as compared to females (40.7%) (p = 0.0067).

**Table 2:** MDR prevalence in an isolated group of microorganisms MDR, multidrug resistant

Organism	MDR Isolates	Non-MDR Isolates	95% CI
*Klebsiella pneumoniae*	30.9% (211/682)	69.1%	27.59-34.51%
*Escherichia coli*	17.8% (31/174)	82.2%	12.84-24.18%

Colistin resistance

Colistin resistance was detected in 3/31(9.67%) of MDR *E. coli *isolates and 1/211 (0.47%) of MDR *Klebsiella pneumoniae *isolates by colistin agar dilution method (Table 3).

**Table 3:** Colistin-resistant isolates in MDR (colistin MIC ≥ 4 µg/mL = resistant; MIC ≤ 2 µg/mL = intermediate/non-resistant) as per the CLSI guidelines CLSI, Clinical and Laboratory Standards Institute; MDR, multidrug resistant

Species	Total Non-MDR Isolates	Total MDR Isolates	Non-Resistant Strains (MIC ≤ 2 µg/mL) (% in Total MDR Isolates)	Colistin-Resistant Isolates (MIC ≥ 4 µg/mL) (% in Total MDR Isolates)	95% CI
*Escherichia coli *(174)	143	31	28 (90.33%)	3 (9.67%)	3.35-24.90%
*Klebsiella pneumoniae *(682)	471	211	210 (99.53%)	1 (0.47%)	0.08-2.64%

Detection of *mcr-1*

The *mcr-1 *gene was identified in one *Klebsiella pneumoniae* isolate obtained from a surgical ICU patient. None of the colistin-resistant *E. coli* isolates tested positive for *mcr-1*. OriginPro 16 software (OriginLab Corporation) was used for data analysis. Given the small expected cell frequencies in the phenotype-genotype comparison, Fisher's exact test was applied and results are expressed as odds ratios (ORs) with 95% CIs. To assess the association between phenotypic colistin resistance and the presence of the *mcr-1* gene, all 242 MDR isolates were classified in a 2×2 contingency table by colistin susceptibility status (resistant vs non-resistant, by agar dilution MIC) and *mcr-1* result (positive vs negative, by RT-PCR). Among colistin-resistant isolates (n = 4), one (25.0%) was *mcr-1* positive; among non-resistant MDR isolates (n = 238), none were tested (all assumed *mcr-1 *negative). Fisher's exact test demonstrated a statistically significant association between phenotypic resistance and *mcr-1 *carriage (p = 0.0165, two-tailed; adjusted OR = 204.4, 95% CI: 7.0-5,945; Haldane-Anscombe correction applied). The wide CI reflects the small absolute event count and should discourage over-interpretation of the effect size. Crucially, *mcr-1 *carriage was identified in only one of four resistant isolates (25.0% sensitivity), indicating that the majority of phenotypic resistance in this sample was not attributable to *mcr-1*. A p-value of <0.05 was considered statistically significant (Table 4).

**Table 4:** Comparison between phenotypic (colistin agar dilution) and genotypic (mcr-1) detection of colistin resistance in MDR isolates MDR, multidrug resistant; MIC, minimum inhibitory concentration

Phenotype	*mcr-1*Positive (n)	*mcr-1*Negative (n)	Total
Colistin-resistant (MIC ≥ 4 µg/mL)	1 (1/4 = 25.0%)	3 (3/4 = 75.0%)	4 (1.65% of MDR)
Non-resistant (MIC ≤ 2 µg/mL)	0 (not tested; assumed negative)	238 (98.35% of MDR)	238
Total	1	241	242

## Discussion

This study provides baseline clinical data on colistin resistance among MDR *Enterobacterales* bloodstream isolates from Central India. The observed prevalence of 1.86% indicates that resistance is emerging in this region, although at lower levels than those reported from certain eastern regions and national datasets [18-20]. Regional variation in reported colistin resistance rates across India likely reflects differences in antibiotic stewardship implementation, laboratory detection capacity, patient case-mix, and the proportion of isolates from critical care settings, rather than true differences in the underlying resistance burden, as also supported by another study of Dwibedy et al. Their research provided comparative data from different states of India, indicating the percentages of colistin-resistant Gram-negative bacterial isolates that caused nosocomial transmission [21]. Apparent similarities or differences between rates from different centers should therefore be interpreted cautiously given the substantial methodological heterogeneity across studies. Only three Indian states - Kerala, Madhya Pradesh, and Delhi - have released action plans aimed at screening for antibiotic resistance as of 2022, according to the data that is currently available.

In our study, a notable finding was the concentration of colistin-resistant isolates in ICUs, and these results are consistent with international trends of antimicrobial resistance (AMR) amplification within critical care [22], suggesting that critical care environments may act as focal points for the selection and transmission of resistant organisms. High antibiotic exposure and prolonged hospitalization are likely contributors to this pattern. Colistin susceptibility was determined by the agar dilution method, which is recommended as a reference method by CLSI and supported by ISO 20776-1:2019. While broth microdilution is the formally defined CLSI reference standard for colistin, agar dilution is widely validated and accepted, particularly in resource-limited settings where automated platforms may not be available. Limitations of the agar dilution method include the potential for colistin adsorption to polystyrene plates and the requirement for careful preparation of cation-adjusted medium. In our study, colistin stock solutions were freshly prepared in distilled water and stored at -20°C; CAMHA was prepared as per NCDC colistin agar dilution for *Enterobacterales *Standard Operating Procedure (SOP) specifications. External validation at NCDC, New Delhi, confirmed our MIC results, strengthening confidence in the methodology. Kar et al. studied colistin resistance among carbapenem-resistant *Enterobacterales* from southern India and did not detect the *mcr-1* gene in any of the resistant isolates tested, suggesting that chromosomal mechanisms may have been responsible for resistance in that cohort. Such clinical isolates may lack the *mcr-1 *gene because of the presence of another *mcr *family gene, or it may be because of the presence of other chromosomal-mediated resistance gene [23]. The identification of a plasmid-mediated *mcr-1* gene in a clinical *Klebsiella pneumoniae* isolate is of particular concern due to its potential for horizontal transfer. Three of four colistin-resistant isolates did not carry the *mcr-1 *gene. The mechanistic basis of resistance in these isolates cannot be determined from the current data. Possible explanations include: (1) other plasmid-mediated colistin resistance genes, *mcr-2* through *mcr-10*, none of which were assessed in this study; (2) chromosomal mutations in *mgrB*, *pmrA*/*pmrB*, or *phoPQ* genes, which are the most commonly reported single-gene chromosomal mechanisms in *Klebsiella pneumoniae*; and (3) other mobile resistance determinants not yet characterized. These three possibilities carry equal evidential weight given the available data, and no mechanistic inference is warranted without whole-genome sequencing or targeted mutation analysis. From a practical perspective, this supports the continued use of phenotypic susceptibility testing as a cost-effective screening strategy in resource-limited laboratories. A study conducted by Bir et al. found the presence of *mcr-1 *gene majorly in *Klebsiella* spp., which is similar to our finding [24]. Adam and Altayb reported that the majority of *mcr-1 *genes were detected among *E. coli *strains. This difference can be explained by the fact that the majority of their isolates were *E. coli* compared to our study, where the majority of isolates were *Klebsiella* spp. [25].

Strengths of the study

This is the first prospective clinical study of colistin resistance in bloodstream isolates from Central India, addressing a recognized surveillance gap. Phenotypic colistin MIC testing by agar dilution was performed on all MDR isolates, and resistant strains were externally validated at a national reference laboratory (NCDC, New Delhi). The two-year prospective design allowed systematic sampling across multiple clinical wards.

Limitations

The present study was conducted at a single tertiary care referral center, which may limit the applicability of the findings to broader community and regional antimicrobial resistance patterns. In addition, the relatively small number of colistin-resistant isolates identified (n = 4) restricted the statistical robustness and generalizability of the prevalence estimates. Molecular analysis in this study was limited to the detection of the *mcr-1* gene, while other plasmid-mediated *mcr* variants (*mcr-2* to *mcr-10*) were not evaluated. Moreover, chromosomal mechanisms implicated in colistin resistance, including mutations in *pmrA*/*pmrB*, *mgrB*, and *phoPQ*, were not assessed. Consequently, the possibility that chromosomal alterations contributed to resistance among the isolates remains hypothetical. The absence of detailed clinical and epidemiological information, such as patient comorbidities, prior antimicrobial exposure, and duration of hospital stay, precluded comprehensive risk-factor analysis. Furthermore, whole-genome sequencing was not performed, limiting in-depth phylogenetic analysis and plasmid characterization of the resistant isolates.

## Conclusions

This prospective study documents the emergence of colistin resistance among MDR *E. coli *and *Klebsiella *spp. causing bloodstream infections in a tertiary care center in Central India. Colistin resistance was detected in 9.67% MDR *E. coli *and 0.47% MDR *Klebsiella pneumoniae *isolates, with resistant isolates predominantly recovered from ICUs. A single plasmid-mediated *mcr-1*-positive *Klebsiella pneumoniae *isolate was identified, representing the first such clinical detection from this region. These findings highlight the importance of ongoing phenotypic surveillance using agar dilution as a cost-effective approach. Furthermore, these observations support a balanced approach that integrates targeted surveillance, pragmatic diagnostics, and context-specific stewardship within existing national antimicrobial resistance frameworks.
